# Our Tangled Family Tree: New Genomic Methods Offer Insight into the Legacy of Archaic Admixture

**DOI:** 10.1093/gbe/evab115

**Published:** 2021-05-24

**Authors:** K D Ahlquist, Mayra M Bañuelos, Alyssa Funk, Jiaying Lai, Stephen Rong, Fernando A Villanea, Kelsey E Witt

**Affiliations:** 1 Center for Computational Molecular Biology, Brown University, Providence, Rhode Island, USA; 2 Department of Molecular Biology, Cell Biology, and Biochemistry, Brown University, Providence, Rhode Island, USA; 3 Brown Center for Biomedical Informatics, Brown University, Providence, Rhode Island, USA; 4 Department of Anthropology, University of Colorado Boulder, Colorado, USA; 5 Department of Ecology and Evolutionary Biology, Brown University, Providence, Rhode Island, USA

**Keywords:** archaic introgression, Neanderthals, Denisovans, human evolution

## Abstract

The archaic ancestry present in the human genome has captured the imagination of both scientists and the wider public in recent years. This excitement is the result of new studies pushing the envelope of what we can learn from the archaic genetic information that has survived for over 50,000 years in the human genome. Here, we review the most recent ten years of literature on the topic of archaic introgression, including the current state of knowledge on Neanderthal and Denisovan introgression, as well as introgression from other as-yet unidentified archaic populations. We focus this review on four topics: 1) a reimagining of human demographic history, including evidence for multiple admixture events between modern humans, Neanderthals, Denisovans, and other archaic populations; 2) state-of-the-art methods for detecting archaic ancestry in population-level genomic data; 3) how these novel methods can detect archaic introgression in modern African populations; and 4) the functional consequences of archaic gene variants, including how those variants were co-opted into novel function in modern human populations. The goal of this review is to provide a simple-to-access reference for the relevant methods and novel data, which has changed our understanding of the relationship between our species and its siblings. This body of literature reveals the large degree to which the genetic legacy of these extinct hominins has been integrated into the human populations of today.

## Introduction

Significance

This review covers ten years of articles published specifically on methods to identify portions of the human genome containing Neanderthal, Denisovan, and even superarchaic ancestry, as well as quantifying the impact of archaic introgression on the human gene pool. In addition, we cover a multitude of articles exploring specific genes, for which archaic versions are thought to have relevant medical consequences. These articles have shown that archaic introgression occurred in Africa as well as Eurasia, that human functional region variation was enriched with an influx of archaic variants fueling natural selection, and that the history of interactions between modern humans and archaic humans is much more complex than was previously thought.

As anatomically modern human (AMH, Box 1) populations began to expand outside of Africa around 50,000–100,000 years before present (YBP, Karmin et al. 2015), they encountered other archaic humans—Neanderthals and Denisovans—and admixture between AMH and these populations left a lasting impact on modern human genomes. Over the past decade, advances in genomic sequencing and detection methods have provided researchers with a better understanding of archaic populations, as well as evidence for multiple admixture events between Neanderthals, Denisovans, and AMH. To date, four high-coverage archaic genomes have been sequenced, three Neanderthal and one Denisovan (Kircher et al. 2012; Prüfer et al. 2014, 2017; Mafessoni et al. 2020), as well as multiple low-coverage Neanderthal and Denisovan genomes (Hajdinjak et al. 2018; Green et al. 2010; Reich et al. 2010; Sawyer et al. 2015; Mafessoni et al. 2020), and the genome of a first-generation offspring between a Neanderthal and a Denisovan (Slon et al. 2018). New evidence also supports admixture with more distantly related “super-archaic” individuals from populations that diverged prior to the split between AMH and Neanderthals-Denisovans, for which we have no direct genome data (Mondal et al. 2019; Ragsdale and Gravel 2019; Wall et al. 2019; Durvasula and Sankararaman 2020; Hubisz and Siepel 2020; Wang, Mathieson, et al. 2020). Various groups of superarchaic humans may have lived at least briefly contemporaneously with Neanderthals, Denisovans, or AMH.

Here, we revisit the most recent literature on archaic admixture in modern human genomes. Our goal is to review the expansion of admixture-related methods and show how newly identified archaic genetic variation has been used to develop a more complex map of archaic admixture between human populations of the past. We discuss in detail the state-of-the-art methods used to identify archaic genome ancestry, and how those methods have allowed us to infer a more complete demographic history of modern humans, particularly in the African continent, and explore functional consequences of archaic introgression in modern humans.

## Big Picture of Archaic Introgression

**Fig. 1.**
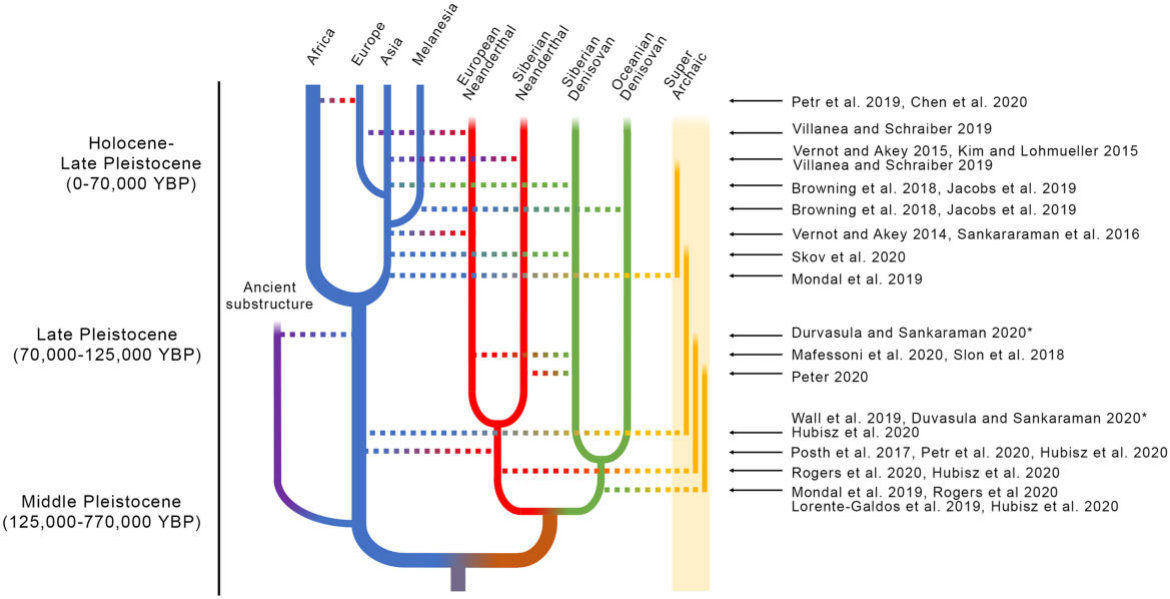
Summary of inferred periods of archaic introgression between anatomically modern humans and archaic humans. Time is represented vertically (but not to scale), with the present time on top, and deep time roughly corresponding to the Holocene-Late Pleistocene, Late Pleistocene, and Middle Pleistocene. Anatomically modern human populations are represented in blue, two Neanderthal populations in red, two Denisovan populations in green, and superarchaic in yellow (this represents one or more populations of hominin that may have contributed to the genome ancestry of modern humans). Possible deep structure in African populations is represented in purple. Horizontal lines indicate gene flow between two populations, but may represent single or multiple gene flow events between the same two populations. Arrows indicate the scientific source which postulates each introgression event. The star notes that ancient African substructure and superarchaic introgression were postulated as alternative hypotheses to explain the same data pattern. It should be noted that in cases where older scientific articles postulated introgression from a population which later came to be understood as separate populations, we assigned the introgression to a specific population, such as European and Siberian Neanderthals, and Oceanian and Siberian Denisovans.

Studies from the last ten years have proposed numerous points of contact and admixture between AMH and archaic humans (fig. 1), describing a complex reticulation of the family tree connecting them. For Neanderthals and Denisovans, sequencing of individuals from each of these archaic populations has provided support for these claims (Green et al. 2010; Reich et al. 2010; Kircher et al. 2012; Prüfer et al. 2014; Sawyer et al. 2015; Prüfer et al. 2017; Hajdinjak et al. 2018; Mafessoni et al. 2020). Most humans carry Neanderthal and Denisovan genome elements, though the amount and type of the contribution varies (Green et al. 2010; Meyer et al. 2012; Sankararaman et al. 2012; Wall et al. 2013; Prüfer et al. 2017; Chen et al. 2020). Perhaps more surprisingly, a number of recent genomic techniques have identified segments of the human genome that appear to originate from unknown populations (Mondal et al. 2019; Ragsdale and Gravel 2019; Wall et al. 2019; Durvasula and Sankararaman 2020; Hubisz and Siepel 2020; Wang, Mathieson, et al. 2020). Some of these events are attributed to human populations that diverged prior to the divergence of Neanderthals and Denisovans, whereas others may be attributed to “ghost populations” of more recently diverged humans for which no direct observation exists. Finally, sequencing of Neanderthal and Denisovan genomes has revealed that admixture was not a unidirectional flow from archaic humans into AMH. Admixture occurred between archaic Neanderthals and Denisovans, and evidence points to the possibility that admixture occurred between those groups and other currently unknown groups as well (Slon et al. 2018; Mafessoni et al. 2020; Peter 2021). Notably, early AMH admixture events contributed prominently to the genomes of Neanderthals (Kuhlwilm et al. 2016; Chen et al. 2020; Hubisz and Siepel 2020). Given the complexity of admixture between these populations, we will consider these groups one at a time starting with Neanderthals—who contribute the largest component of archaic ancestry in modern genomes, then Denisovans—who contribute the second largest component with the widest geographic distribution, and finally, evidence for various superarchaic human groups—that admixed into AMH, Neanderthals, and Denisovans.

### Neanderthal and AMH Admixture

When AMH dispersed outside of Africa, they encountered and admixed with Neanderthals (Green et al. 2010). Neanderthals occupied a vast area of Asia and Europe at the time AMH dispersed first into the Middle East ∼75,000 YBP, and later Europe and Asia ∼47–55,000 YBP (Karmin et al. 2015; Poznik et al. 2016; Skoglund and Mathieson 2018). Moreover, the size distribution of Neanderthal segments in modern human genomes is indicative of a time-frame for admixture of 50,000–60,000 YBP (Sankararaman et al. 2012; Moorjani et al. 2016; Skoglund and Mathieson 2018), which is prior to the diversification of East Asian and European lineages. The genome of Ust'-Ishim, an ancient AMH of equidistant relation to modern East Asians and Europeans, has Neanderthal ancestry at similar levels to modern Eurasians, but found in longer haplotypes, consistent with an admixture episode occurring ∼52,000–58,000 YBP (Fu et al. 2015; Moorjani et al. 2016).

The Neanderthal component of the modern human genome is ubiquitous in non-African populations, and yet is quantitatively small, representing on average only ∼2% of those genomes (Prüfer et al. 2017; Green et al. 2010). This pattern of Neanderthal ancestry in modern human genomes was initially interpreted as evidence of a single period of admixture, occurring shortly after the out-of-Africa (OOA) bottleneck (Green et al. 2010; Sankararaman et al. 2012). However, subsequent research showed that Neanderthal ancestry is higher by an additional ∼8–20% in modern East Asian individuals relative to modern European individuals (Meyer et al. 2012; Wall et al. 2013; Prüfer et al. 2017; Chen et al. 2020). Given this observation, it is also possible that admixture occurred multiple times; the first pulse of Neanderthal gene flow into the population ancestral to East Asians and Europeans could have been supplemented by additional pulses into East Asians after both populations had diverged (Vernot and Akey 2015; Vernot et al. 2016; Villanea and Schraiber 2019). An updated study has indicated that some Neanderthal ancestry in modern Europeans was previously undetected as an artifact of modern gene flow between European and African populations, the latter of which were used as outgroups in the original studies (Chen et al. 2020). This correction puts the excess of Neanderthal ancestry in East Asians over Europeans at the lower end of the range, at 8%, but it doesn’t fully explain the discrepancy in Neanderthal ancestry components between East Asian and European populations.

Thus, currently the most parsimonious model is that of admixture occurring multiple times; the main pulse of Neanderthal gene flow into the population ancestral to East Asians and Europeans was supplemented by additional gene flow after both populations had split. This model is supported by the recent discovery of multiple Neanderthal variants co-occurring in modern human populations, some presenting geographic patterns consistent with independent admixture between modern humans with European and Siberian Neanderthal subgroups (Taskent et al. 2020; Zeberg et al. 2020; Villanea et al. 2021). This pattern is somewhat confused by later AMH population expansions and replacement, as evidenced by ancient European individuals from 42,580 to 45,930 years ago presenting immediate Neanderthal ancestry (∼6 generations back), but who are more closely related to modern East Asians than the modern European populations that later replaced them (Hajdinjak et al. 2021).

Alternative explanations propose the differences in the level of Neanderthal ancestry in East Asian and European populations could be the result in the differing demographic histories between the populations. The elevated Neanderthal ancestry in East Asian individuals could be explained by their lower ancestral effective population size relative to Europeans, which would reduce the efficacy of purifying selection against deleterious Neanderthal alleles (Sankararaman et al. 2014; Harris and Nielsen 2016). However, two studies (Kim and Lohmueller 2015; Juric et al. 2016) found that differences in the strength of purifying selection and population size are unlikely to explain the enrichment of Neanderthal ancestry in East Asian individuals. Most recently, a new hypothesis has suggested that East Asians have a slightly but significantly longer generation time than Europeans, which would decrease the loss of Neanderthal ancestry to genetic drift due to there being fewer generations since archaic introgression in East Asians than Europeans (Coll Macià et al. 2021).

Recent work has demonstrated that Neanderthals may have also inherited genetic variants from AMH (Kuhlwilm et al. 2016; Chen et al. 2020; Hubisz and Siepel 2020). For example, although early Neanderthals—such as the individual from Sima de los Huesos dated to ∼430,000 YBP—have mitochondrial genomes (mitogenomes) that are similar to that of Denisovans (Meyer et al. 2016), the mitogenomes of late Neanderthals and modern humans are less molecularly divergent than the rest of their genomes. Posth et al. (2017) observed that although the population divergence time between Neanderthals and AMH is estimated as 765–550,000 YBP, the corresponding divergence time for mtDNA has been dated to only ∼400,000 YBP. It has been suggested that gene flow prior to ∼270,000 YBP from an AMH African source resulted in a replacement of mitogenomes in Late Pleistocene Neanderthals. Similarly, although the Denisovan and Neanderthal lineages diverged from AMH around 700,000 YBP, late Neanderthal and AMH Y chromosomes have a much shorter divergence time of around 370,000 YBP (Petr et al. 2020). In contrast, the divergence time of Denisovan and AMH Y chromosomes is concordant with the rest of the genome. Finally, AMH introgression, possibly from an early out of Africa dispersal, has been detected in the Neanderthal autosomal genome as well (Kuhlwilm et al. 2016; Chen et al. 2020), and may comprise as much as 3% of the Neanderthal genome (Hubisz and Siepel 2020). Together, this provides strong evidence that gene flow from AMH to Late Pleistocene Neanderthals has occurred multiple times.

### Denisovan and Modern Human Admixture

In 2010, Reich et al. (2010) revealed that molars and finger bones, at first thought to be Neanderthal in origin, had yielded the genome of a yet unidentified archaic hominin, dubbed Denisovans after the cave in which the remains were found. Surprisingly, although the only anatomical remains currently attributed to Denisovans have been recovered from Siberia and the Tibetan Plateau, the greatest proportion of Denisovan admixture in AMH has been found in Melanesia (Reich et al. 2010; Meyer et al. 2012), with substantial admixture also in Southeast Asia and parts of East Asia, hinting at a vast distribution of Denisovans in mainland Asia (Vernot and Akey 2015; Sankararaman et al. 2016).

Investigating the variation in Denisovan genome fragments found in AMH genomes revealed that Denisovans were more genetically diverse than Neanderthals, had deep population structure, and that as many as three distinct admixture events from Denisovans into AMH could be identified (Browning et al. 2018; Jacobs et al. 2019). Furthermore, although Denisovan admixture was assumed to be nearly zero in Europeans, a recent study revealed small traces of Denisovan ancestry in Icelanders. These traces of Denisovan ancestry are better explained through direct admixture between Denisovans and the Eurasian ancestors of modern European populations, as opposed to more recent gene flow between Asian and European populations (Skov et al. 2020). Thus, although fossil evidence on the complete geographic range of Denisovans remains elusive, the geographic distribution and genetic diversity of Denisovan genome introgression points to distinct lineages of Denisovans in East Asia and in Melanesia, and perhaps even further west, each encountering and admixing with modern humans at different geographic locations. For example, the genomes of two ancient AMH from East Asia—Salkhit and Tianyuan—provide direct evidence that AMH who lived in East Asia 40,000 YBP had already met and admixed with Denisovans, and that this Denisovan ancestry was distinct from the Denisovan ancestry carried by Melanesians (Massilani et al. 2020). Likewise, ancient protein analysis from a mandible, and environmental DNA extractions from the soil of the Baishiya Karst Cave, point at a long term Denisovan presence in the Tibetan plateau (Zhang, Xia, et al. 2020), which could have been the donor population for the famous Denisovan high-altitude adaptation haplotype found in modern Tibetans (Huerta-Sánchez et al. 2014; Racimo, Gokhman, et al. 2017; Zhang et al. 2021).

### Superarchaic Populations and Modern Human Admixture

The advent of methods that do not require archaic references for detecting introgression have revealed portions of the human genome that may derive from sources more divergent than even the common ancestor of AMH, Neanderthals, and Denisovans, dubbed “super-archaic” humans. Interestingly, there is both evidence of superarchaic admixture events outside of Africa (Mondal et al. 2019; Hubisz and Siepel 2020) as well as within Africa. The geographic range of admixture events could point to gene flow from hominin species, such as *Homo antecessor* or *Homo erectus*, for which there is fossil evidence of a wide geographic range across both the African and Asian continents (Antón 2003; Bergström et al. 2021).

Evidence for superarchaic introgression outside of Africa has been found using independent methods (Mondal et al. 2019; Hubisz and Siepel 2020). Mainland Asian and Oceanian populations show evidence of introgression from an unknown extinct archaic hominin population that is likely closely related to the Neanderthal-Denisovan clade (Mondal et al. 2019). Introgression from unknown sources is not limited to AMH populations—Denisovans, as well as the population ancestral to Neanderthals and Denisovans, show signals of introgression from superarchaic human populations (Prüfer et al. 2014; Rogers et al. 2020). As much as 1% of the Denisovan genome may derive from these unknown sources, with up to 15% potentially passed along to AMH through Denisovan admixture (Hubisz and Siepel 2020).

Based on the geographic distribution of Neanderthal and Denisovan fossil evidence, it had been thought that African populations did not experience archaic introgression from these sources, which prompted the use of African populations as outgroups to detect genomic introgression. Methods that do not use Africans as an outgroup have now inferred genetic contributions from unknown human groups found in sub-Saharan African genomes (Lorente-Galdos et al. 2019; Wall et al. 2019; Chen et al. 2020; Durvasula and Sankararaman 2020). Because of the novelty and importance of this discovery, we explore this topic, and related questions of demography within the African continent, in its own section below (see “New Insights on the Demography of Archaic African Populations”).

The Southeast Asian Islands is another geographic area where there could be potential genomic contributions from other superarchaic species, such as *Homo luzonensis* and *Homo floresiensis*, given that fossil temporal ranges overlapped with the expansion of AMH into Oceania and the Pacific Islands (Teixeira et al. 2021). However, two recent studies into the genomic composition of individuals from the Southeast Asian Islands (Tucci et al. 2018; Teixeira et al. 2021) found no evidence of archaic introgression beyond Neanderthal and Denisovan ancestry. It is worth noting that Denisovan ancestry increases in a marked gradient between mainland Southeast Asia and Oceania, indicating a complex and long-lasting interaction between AMH and Denisovans (Choin et al. 2021).

As the evidence for admixture with various superarchaic human populations continues to build in the future, there is the lingering question of the identity of the various populations which may have interbred with AMH, Neanderthals, and Denisovans. In some cases, signals of superarchaic introgression may turn out to be the result of structure or demographic events occurring within known populations, rather than evidence of unknown or unsampled populations. Although finding hominin remains predating the divergence of Neanderthals and Denisovans with intact aDNA for ancient genome sequencing is unlikely, the best bet given current technology might be the use of proteomic technology, which in coming years will provide protein sequence information from hominin bones older than the maximum antiquity of aDNA preservation (Welker 2018; Chen et al. 2019). Proteomic data from a mandible bone found in Tibet, predicted to be 160,000 years old, were used in a phylogenetic analysis which demonstrated that the bone most closely resembles Denisovan samples (Chen et al. 2019). Although there are limits to the collection and application of proteomic data, ancient proteomes can be processed to find “single amino acid polymorphisms”: heritable units analogous to single nucleotide polymorphisms in DNA, and used to conduct genetic analyses of samples. Proteomic data are especially promising for samples like the one described in Chen et al. (2019), where age and condition of the sample preclude DNA extraction and analysis. For further review of this topic, see Welker (2018) and Muth et al. (2018). For a review of proteomics with applications to remains and fossils over 1 million years old, see Schweitzer et al. (2019).

### Admixture between Archaic Humans

Any review of the complex pattern of hominin admixture would be remiss not to discuss the extraordinary finding of the Denisovan-11 individual. Denisovan-11, or Denny, was a first-generation hybrid, the offspring of a Neanderthal mother and a Denisovan father, found in the Denisova cave in Siberia (Slon et al. 2018). Furthermore, Denny’s Neanderthal ancestry was more closely related to European Neanderthals than to other Siberian Neanderthals, some of which were also found in older fossil layers at Denisova cave. This pattern indicates that the European and Siberian Neanderthal lineages, while genetically distinct, were not always isolated geographically (Mafessoni et al. 2020), with further sedimentary aDNA evidence from Galeria de las Estatuas cave in Spain suggesting multiple radiation events of Neanderthal populations (Vernot et al. 2021). To add more complexity to this unique finding, at least five long segments (∼1 Mb) of Denny’s genome carry Neanderthal alleles on both chromosomes, suggesting that the Denisovan father’s lineage presented Neanderthal introgression deep in its past. This pattern of long-term allele sharing between Denisovans and Siberian Neanderthals is also reported in Peter (2021), suggesting that Denisovan-Siberian Neanderthal admixture occurred continuously through most of the Middle Paleolithic, although European Neanderthals do not show this pattern of Denisovan introgression. This is an important perspective when considering the complexity of modern human genomic admixture, as simple models involving unidirectional gene flow from one species to another are insufficient, and future demographic models will be required to include additional degrees of reticulation.

## Novel Methods for Detecting Archaic Admixture

Novel and improved methods to detect archaic admixture and localize archaic ancestry segments (sometimes also referred to as archaic introgression and introgressed segments, respectively) have rapidly become available over the last decade. In 2015, Racimo et al. reviewed existing methods to detect archaic introgression, including Patterson’s *D* (Green et al. 2010; Durand et al. 2011), the S* method (Wall 2000; Plagnol and Wall 2006; Racimo et al. 2015), and phylogenetic evidence (Mendez et al. 2012). They also considered methods to localize introgressed segments, such as applying S* in local windows (Vernot and Akey 2014), and statistical models, such as Hidden Markov Models (HMMs, Prüfer et al. 2014) and Conditional Random Fields (CRF, Sankararaman et al. 2014).

**Fig. 2.**
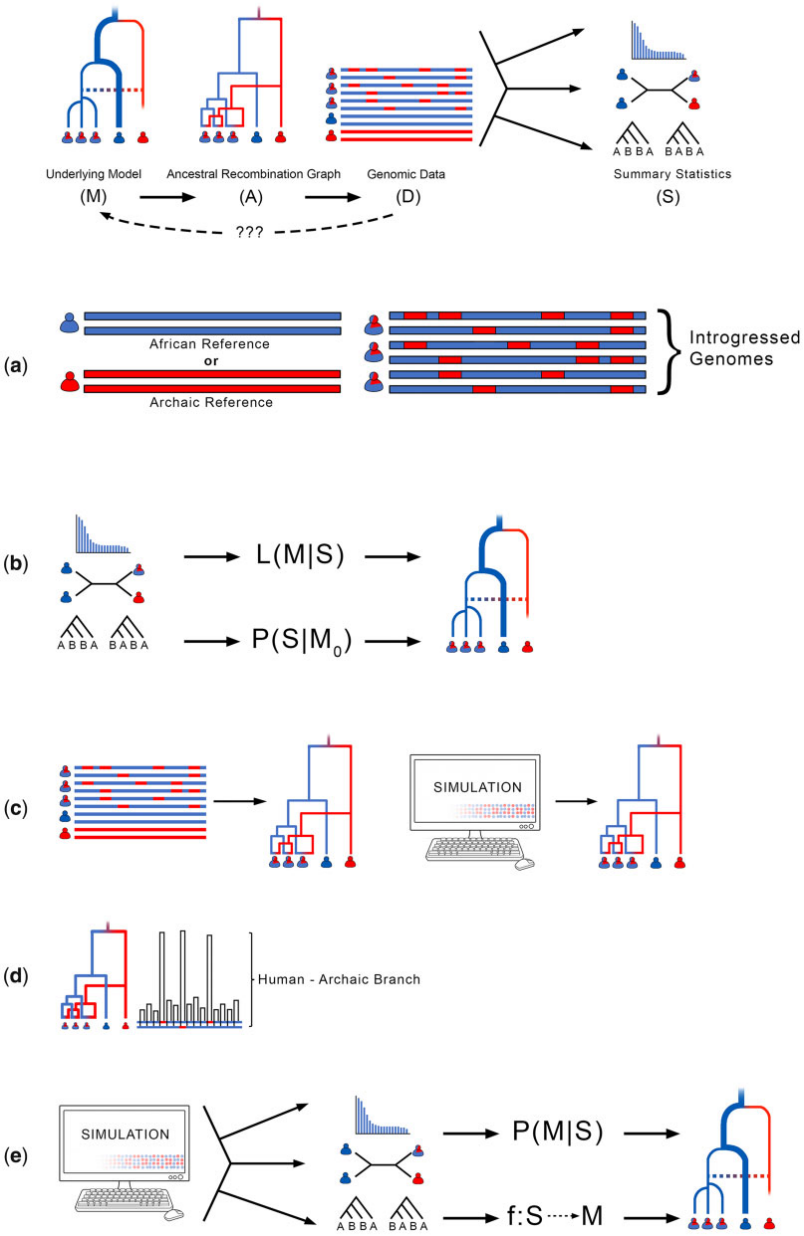
Classification of different methods for genome-wide inference of archaic introgression. An underlying but unknown model (M) gives rise to a pattern of mating and reproduction that can be represented by a data structure, such as an ancestral recombination graph (A), for which we can observe genomic data (D). The information in D can further be simplified by calculating summary statistics (S). Our objective is to gain information about M. In practice, this objective is approached in a number of ways: (*a*) Comparison of genome variation using either archaic or unadmixed (usually African) reference genomes. (*b*) Using summary statistics (S) to compute the likelihood (L) under M, or the probability (P) of S under alternate models M_0_. Computing S from D summarizes salient information about A, for which is possible to make inferences about M using null hypothesis testing (*D*-statistics), Maximum Likelihood Estimation (SFS, LD), or Bayesian inference (gene tree methods). (*c*) Attempting to infer ARG (A) directly from D, or from simulations (ARGweaver, Relate, and tsinfer). (*d*) Using the ARG or simulated ARG to predict introgressed branch lengths. Predictions about coalescent wait times informed from A are used to classify genome segments (ARGweaver-D). (*e*) Simulating data summaries (S), which could be user-defined statistics (Approximate Bayesian Computation) or automatically learned summaries (Machine Learning). Here, many mappings of M to S are generated from the simulations, and used to learn an inverse mapping from S to M in empirical data. This applies to ABC (P(M|S)) or ML (f: S→M).

Here, we will focus on two categories of methods: 1) methods that have innovated around data needs, including relaxing the need for reference or outgroup data (fig. 2*a*), and 2) methods that take advantage of computational advances to provide new opportunities for detecting archaic admixture and localizing archaic ancestry segments (fig. 2*b*–*e*).

### Methods That Have Innovated around Data Needs

Innovative methods have solved problems associated with the need for reference or outgroup data, allowing us to: 1) localize ancestry segments without an archaic genome as a reference, and 2) detect or localize archaic ancestry segments without using African populations as an outgroup.

#### Localizing Ancestry Segments without Archaic Reference Genomes

Ancestry from unknown sources, including superarchaic human populations as well as yet unsampled AMH populations, dubbed “ghost populations,” remains more difficult to study because the only known evidence for these events is contained in the human genome. Fortunately, method development in the last five years have presented us with many methods (table 1) capable of detecting archaic admixture and localizing ancestry segments without relying on archaic reference genomes. These include the aforementioned S* (Plagnol and Wall 2006; Vernot and Akey 2014; Vernot et al. 2016) and *Sprime* methods (Browning et al. 2018), which look for long, linked, diverged tracts characteristic of archaic ancestry segments; *VolcanoFinder* (Setter et al. 2020), which looks for a volcano-shaped genetic diversity footprint characteristic of adaptively introgressed loci; Archaic Introgression Explorer (*ArchIE*, Durvasula and Sankararaman 2019), which combines population genetic summary statistics to look for archaic introgression loci; and HMMs, which can be used to detect archaic introgression in the absence of archaic genomes by looking for segments with a high density of variants unseen in an unadmixed outgroup (Skov et al. 2018; Peter 2021). Methods for inferring demographic models with complex gene flow have also improved in recent years. These include model selection using the conditional site frequency spectrum (Durvasula and Sankararaman 2020) and site pattern frequencies (Rogers et al. 2020); phylogeny estimation from gene trees under demographic models with gene flow (Gronau et al. 2011; Kuhlwilm et al. 2016; Hey et al. 2018); and improved demographic parameter inference using two-locus moment statistics (Ragsdale and Gravel 2019). These novel inference methods have allowed for a broader consideration of demographic models with gene flow from superarchaic and ghost populations.

**Table 1:** Methods for Detecting and Identifying Archaic Admixture

Method	Publication	Link
Admixfrog	Peter (2021)	https://github.com/BenjaminPeter/admixfrog
ArChie	Durvasula and Sankararaman (2019, 2020)	https://github.com/sriramlab/ArchIE
Conditional Random Field (CRF)	Sankararaman et al. (2014, 2016)	
Conditional Site Frequency Spectrum (CSFS)	Durvasula and Sankararaman (2020)	
ARGWeaver-D	Hubisz et al. (2020)	https://github.com/CshlSiepelLab/argweaver
Convolutional Neural Network (CNN)	Flagel et al. (2019)	https://github.com/flag0010/pop_gen_cnn
	Gower et al. (2021)	https://github.com/grahamgower/genomatnn
diCal-admix, CSD	Steinrücken et al. (2018)	http://dical-admix.sourceforge.net
HMM	Skov et al. (2018)	https://github.com/LauritsSkov/Introgression-detection/
IBDmix	Chen et al. (2020)	https://github.com/PrincetonUniversity/IBDmix
IMa3	Hey et al. (2018)	https://github.com/jodyhey/IMa3
Legofit	Rogers (2019)	https://github.com/alanrogers/legofit
Moments, moments. LD	Ragsdale and Gravel (2019)	https://bitbucket.org/simongravel/moments/src/master/
Relate	Speidel et al. (2019)	https://myersgroup.github.io/relate/
R_D_, U, and Q95 Statistics	Racimo et al. (2017)	
S*	Plagnol and Wall (2006); Racimo et al. (2015), Vernot and Akey (2014), Vernot et al. (2016)	https://github.com/bvernot/freezing-archer
SPrime	Browning et al. (2018)	https://github.com/browning-lab/sprime
tsinfer	Kelleher et al. (2019)	https://github.com/tskit-dev/tsinfer
VolcanoFinder	Setter et al. (2020)	http://degiorgiogroup.fau.edu/vf.html

Note.—The publication where each method is described is given. Where available, links to code repositories are also provided.

#### Detecting Archaic Admixture without an African Outgroup

Using African populations as an “unadmixed” outgroup has enhanced the ability of researchers to detect admixture between Neanderthals or Denisovans and non-African AMH populations (fig. 2*a*). However, this assumption prevents the detection of archaic admixture events that occurred within Africa, and underestimates archaic ancestry in portions of the genome where archaic alleles are shared between African and non-African populations. Several novel methods avoid this shortcoming, which has allowed for the discovery of archaic ancestry segments in African populations. Some of these methods, such as *ArchIE* (Durvasula and Sankararaman 2019; 2020), enable another population to be used as an outgroup to allow for the discovery of introgression in African populations, whereas others, such as *VolcanoFinder* (Setter et al. 2020), *IMa3* (Hey et al. 2018), and *IBDMix* (Chen et al. 2020), do not require the assumption of an unadmixed outgroup. *VolcanoFinder* identifies introgressed segments that have been the subject of positive selection by looking for a characteristic pattern of excess intermediate-frequency polymorphism creating a “volcano” shape in plots of pairwise genetic diversity. *IBDMix* estimates identity-by-descent (IBD) probabilities between modern genomes and an archaic reference genome to localize archaically introgressed segments without the need for an unadmixed modern human outgroup. *IMa3* does not localize archaic ancestry segments, but instead approximates the probability of demographic and genealogical parameters given the observed data, using certain summary statistics calculated on the data (fig. 2*b*). The result is that *IMa3* is able to demonstrate that admixture is necessary to explain the patterns observed in modern samples without the need for outgroup populations. Finally, cross-coalescent analysis based on the sequential Markovian coalescent can also be applied between African genomes and an archaic genome to identify archaic ancestry in Africa due to back-migration from Eurasia (Bergström et al. 2020).

#### Other Advances in Available Data for Inferring Archaic Introgression

Methods that still rely on archaic genomes or African outgroups have also been updated and refined (Vernot and Akey 2014; Vernot et al. 2016; Browning et al. 2018; Skov et al. 2018; Racimo, Marnetto, et al. 2017; Peter 2021; Skov et al. 2020), leveraging an increase in the number and diversity of modern genomes available (1000 Genomes Project Consortium et al. 2015; Mallick et al. 2016; Malaspinas et al. 2016; Bergström et al. 2020). Sequencing of additional archaic genomes (Prüfer et al. 2017; Mafessoni et al. 2020) has provided a powerful supplement to methods to detect archaic admixture (Browning et al. 2018). Computational advances have also allowed researchers to incorporate larger data sets with additional samples for a fuller picture of admixture events. For example, S* did not originally identify introgressed genome segments (Wall 2000; Plagnol and Wall 2006; Racimo et al. 2015), but a refined version of S* is now able to localize introgressed segments (Vernot and Akey 2014; Vernot et al. 2016; Browning et al. 2018). By comparing these putative introgressed sequences with new Neanderthal or Denisovan genomes, researchers have been able to better estimate the amount of archaic ancestry and number of admixture events from these groups (Browning et al. 2018).

### Computational Advances Relating to Detecting Archaic Admixture

These include: 1) methods related to the Ancestral Recombination Graph (ARG) and genealogical inference; and 2) methods that make use of machine learning methods and approximate Bayesian computation (ABC).

#### The ARG and Genealogical Inference

Methods related to the ARG have expanded significantly in the last few years. Theoretically, inferring the ARG means inferring the complete genealogical history, including recombination and coalescence, for every piece of the genome for all sampled individuals (fig. 2*c*). Full knowledge of the ARG would provide all the information available in a set of genomes about the history of those lineages, including events such as admixture, demographic changes, and recombination. An ARG also may also reveal information about selection and adaptation over time. In practice, tracking the history of each recombinant fragment, and storing such a large amount of information, is a herculean task. As such, all of the ARG-based methods discussed below make use of some simplifications or heuristic approaches to provide approximations of the full theoretical ARG. *ARGWeaver* (Rasmussen et al. 2014) allows for ARG inference with a sample size of tens of mammalian genomes. *ARGWeaver-D* (Hubisz et al. 2020) builds on the *ARGWeaver* model, allowing for tracing the origin of genomic segments through the inferred ARG under a user-specified demographic model, and allowing the user to include heterochronous samples. The ability to trace the origin of genomic segments allows *ARGWeaver-D* to ascribe specific ancestry to genomic segments in modern human genomes, as well as in Neanderthal and Denisovan genomes, even identifying portions of the Denisovan genome which originated from an unknown, superarchaic human population. However, applying *ARGWeaver-D* is very computationally expensive, and the complexity of demographic models that can be considered is limited. *ARGWeaver-D* also can only be applied to tens of individuals at most.

Additional methods give insight into the ARG while taking advantage of simplifications that allow for scalability and computational efficiency. *Relate* (Speidel et al. 2019) presents an efficient method to produce genealogies for each site in the genome. *Relate* first constructs genealogical trees at each site, building on the HMM process described by Li and Stephens (2003). Coalescent times are then inferred in a separate process using an iterative Markov Chain Monte Carlo algorithm. The process is designed to scale to over ten thousand individuals. Using a different set of simplifications based on the Li and Stephens model, *tsinfer* (Kelleher et al. 2019) leverages the tree sequence data structure, a method that can efficiently record genealogical trees for each genomic site, by recognizing that genealogies at adjacent genomic sites are highly correlated. The tree-sequence encoding stores edges of adjacent, correlated trees just once, allowing for efficient storage of information, and enabling fast calculation of many tree-sequence features, and scaling to over a hundred thousand individuals. Although tree sequences provide a computationally efficient approximation of much of the information contained within the complete ARG, the model makes simplifying assumptions, including a single origin for mutation (no recurrent mutations or back mutation), and an assumption that variant age can be approximated by variant frequency. Tree sequence construction is also vulnerable to errors in phasing and sequencing, and requires high quality phased data as an input. Both *Relate* and *tsinfer* can be used to detect archaic admixture in large population genomic data sets by identifying sites in the genome with exceptionally long branch lengths or long time to the most recent common ancestor (TMRCA) in the ARG (fig. 2*d*). Additional data from archaic genomes can allow for discovery and validation of archaic ancestry segments. These methods also have the potential to more explicitly incorporate heterochronous sampling as part of the process of constructing a complete population history. Forthcoming extensions of these methods allow for inclusion of ancient and archaic samples to improve estimation of genealogies and the timing of events (Speidel et al. 2021; Wohns et al. 2021).

#### Machine Learning and ABC

Machine learning methods and ABC have become prominent features in recent publications on the detection of archaic admixture. Both methods take advantage of fast, efficient software for population genetic simulation (Kelleher et al. 2016; Haller et al. 2019) to sample model parameters from a prior distribution (fig. 2*e*). Both machine learning and ABC use the information from simulations to infer population genetic parameters that fit the genomic data. ABC is a likelihood-free inference method that uses summary statistics as input. Summary statistics from genomic and simulated data are compared to find the combination of simulated parameters that yield simulated summary statistics that are closest to the summary statistics of the genomic data. If the distance between the genomic and simulated summary statistics is below a predefined tolerance, the model parameters are accepted. Otherwise they are rejected, but the closest model parameters are used to update additional rounds of simulation (Villanea et al. 2020). However, ABC is usually based on hand-selected summary statistics, and often requires substantial investment in computational resources (usually >10^6^ individual simulations) to perform accurate parameter inference (Beaumont et al. 2002; Sousa et al. 2009).

Supervised machine learning also takes advantage of fast, efficient methods for population genetic simulation to find the population parameters that produce simulated data most similar to the observed data set (fig. 2*e*). In supervised learning, simulated data are partitioned into training and test sets, and a variety of learning algorithms are used to classify the data and make inferences (for a comparison of different learning algorithms, see Caruana et al. 2008). Supervised learning can be applied in a genome scan approach to localize archaic introgression (Gower et al. 2021). *ArChie* (Durvasula and Sankararaman 2019) uses logistic regression on a preselected set of summary statistics to distinguish AMH-derived haplotypes from those that derive from other archaic human populations. Similarly, *FILET* (Schrider et al. 2018) uses the extra trees classifier on a preselected set of summary statistics to identify introgressed loci (fig. 2*e*). Inference directly from sequence data, without the need for summary statistics, may also be possible in future work (Chan et al. 2018; Flagel et al. 2019). Supervised learning can also be applied to perform demographic model selection. Villanea and Schraiber (2019) used deep learning to match summaries of observed data with models that consider either single or multiple archaic admixture events. Supervised machine learning often requires a fraction of the simulations used in ABC. However, supervised machine learning does not usually provide inference of meaningful posterior probabilities.

To ease these shortcomings, the advantages of both ABC and supervised machine learning have been combined in recent work (*ABC-DL*, Lorente-Galdos et al. 2019; Mondal et al. 2019). Their goal was to reduce the volume of simulations required by letting supervised learning produce refined summary statistics that maximize information from fewer simulations, and then used these refined summary statistics in ABC to infer posterior distributions. This approach also negates a major weakness of supervised machine learning, as it allows for the quantification of uncertainty through the inference of posterior probability distributions.

The advances represented by these methods have revolutionized our understanding of archaic admixture at a rapid pace (for a summary, see table 1, fig. 2). In the near future, we predict significant expansions of both data sources and methods, which will open new lines of inquiry and give new insight into the legacy of archaic admixture (see Conclusions for further discussion). Next, we focus on two areas where novel results have already changed our biological understanding in the last decade: Demographic models of human ancestry in the African continent; and clarifying the effects of functional regions influenced by archaic alleles.

## New Insights on the Demography of Archaic African Populations

Methods that do not require archaic reference genomes or an African outgroup population have also enabled further exploration of archaic admixture in Africa, where many superarchaic hominin groups are known to have lived, but where timing and conditions likely preclude the discovery of ancient DNA sources. These advances allow for the identification of archaic admixture from populations that have not been genetically sampled, some of which may have lived and interacted with humans far earlier than Neanderthals or Denisovans.

Some of the earliest studies of archaic introgression also identified signals of archaic gene flow into African populations. The S* statistic has been used to detect signals of archaic admixture with Yorubans (Plagnol and Wall 2006; Wall and Hammer 2006; Wall et al. 2009) and Pygmy hunter-gatherer populations (Lachance et al. 2012; Hsieh, Veeramah, et al. 2016), and multiple gene haplotypes were identified in African individuals that show deep divergence times compared with haplotypes found in other human populations (700,000–1.98 million YBP, Garrigan et al. 2005; Hammer et al. 2011). However, it is also possible that this signal of archaic introgression into Africans instead reflects deep population structure within human populations (see Skoglund et al. 2017).

Applying these new methods to African populations has led to the identification of multiple unknown hominin sources of introgression, potentially including superarchaic humans and ghost populations of AMH. However, there is not yet a consensus on the timing and demographic features of the admixture events (Lorente-Galdos et al. 2019; Durvasula and Sankararaman 2020). Gene flow from unknown hominin sources into AMH may have occurred as recently as 30,000 YBP (Hsieh, Woerner, et al. 2016), but subsequent studies find the signal for archaic admixture at a much earlier time (Lorente-Galdos et al. 2019; Durvasula and Sankararaman 2020). Some studies suggest that West Africans must have had gene flow from a human population that diverged before the human-Neanderthal split (Durvasula and Sankararaman 2020), but it’s also possible that the archaic population diverged from AMH or Neanderthals after the Human-Neanderthal split and admixed with both African and non-African populations (Hey et al. 2018; Ragsdale and Gravel 2019).

New demographic methods also allow for a more accurate and detailed reconstruction of African demographic history. Modern African populations are a mix of Southern and Eastern African hunter-gatherer, East African pastoralist, and West African agricultural groups which admixed over the past 10,000 years (Skoglund et al. 2017; Henn et al. 2018; Wang, Mathieson, et al. 2020; Sengupta et al. 2021). The ancestors of hunter-gatherers and farmers lived about 90–150 thousand YBP (Hsieh, Veeramah, et al. 2016), whereas Eastern and Southern hunter-gatherers within the Khoisan (known as click languages) language family diverged at least 30,000 YBP (Tishkoff et al. 2007; Pickrell et al. 2012). Modern populations with larger amounts of hunter-gatherer ancestry, including Pygmy populations from Central Africa and the Mbuti and San peoples, show stronger signals of archaic introgression as well as deeper population divergence times (Lachance et al. 2012; Pickrell et al. 2012; Hsieh, Veeramah, et al. 2016). Ancient hunter-gatherer groups in sub-Saharan Africa have been geographically isolated with limited admixture for a long time (Wang, Goldstein, et al. 2020), and may have diverged around 160–350 thousand YBP (Schlebusch et al. 2017; Schlebusch and Jakobsson 2018; Fan et al. 2019). Additionally, some regions of the genome in African populations including Yorubans and the San show even deeper divergence times, >1 million years, which likely reflect archaic admixture (Garrigan et al. 2005; Speidel et al. 2019; Wang, Mathieson, et al. 2020). Different African populations show different levels of admixture and archaic ancestry (Ragsdale and Gravel 2019; Wang, Mathieson, et al. 2020), and the distribution of these segments is not consistent with a single pulse of archaic admixture (Wang, Mathieson, et al. 2020), suggesting that these signals reflect multiple admixture events through time, possibly with multiple populations of archaic humans.

When considering archaic admixture in Africa, it is also important to have a clear demographic model of admixture both before and after the OOA event. Back-migration from Eurasia into Africa post OOA and post-Neanderthal introgression likely introduced not just Eurasian ancestry into Africa (Henn et al. 2012; Pagani et al. 2012; Hodgson et al. 2014; Pickrell et al. 2014; Petr et al. 2019), but also Neanderthal ancestry (Sánchez-Quinto et al. 2012). Furthermore, a ∼4,000-year-old ancient Ethiopian genome (Gallego Llorente et al. 2015) confirms that back migration was occurring after ∼4000 YBP, but it may have also been occurring as early as the European-East Asian split (Chen et al. 2020), or even concurrent with or immediately following the OOA event (Cole et al. 2020).

In summary, novel methods and data sources have revealed a much more complex view of African human demographic history than was previously considered. Our inference of admixture in human populations goes beyond the archaic genomes that have been sequenced: We have the ability to identify previously unknown sources of admixture, possibly from superarchaic hominins who lived much earlier than Denisovans or Neanderthals or from ghost populations of AMH. Multiple hominin species overlapped with one another temporally and geographically in Africa (Herries et al. 2020), and some species, such as *H. erectus* and *H. antecessor* lived for thousands of years and were broadly distributed geographically (Carotenuto et al. 2016; Rizal et al. 2019; Bergström et al. 2021), making admixture with these hominins possible. Signatures of ghost populations have been detected in other primates as well, although not in all species (Hey et al. 2018; Kuhlwilm et al. 2019). It is important to consider that other complex demographic events may contribute to these signals. For example, population replacement events have been demonstrated to be common throughout human history (de Barros Damgaard et al. 2018; Lipson et al. 2018; Posth et al. 2018; Mathieson and Scally 2020), and even occurred in Neanderthals (Meyer et al. 2016; Slon et al. 2018). Additional genomes from archaic humans, and AMH individuals living in the past, will be necessary to clarify what these signals represent.

## Functional Consequences of Archaic Introgression

Archaically introgressed haplotypes may not be neutrally evolving, but instead harbor functional alleles with tissue-specific phenotypic consequences that impact fitness, and thus were likely subject to both purifying and positive selection. There is evidence that Neanderthals had a small population size, reducing the effectiveness of purifying selection and allowing Neanderthal genomes to accumulate more deleterious alleles relative to AMH (Castellano et al. 2014). Once admixture occurred between Neanderthals and AMH, deleterious alleles from Neanderthals would experience increased pressure from purifying selection due to a larger population size. Neanderthal haplotypes are reduced in genomic regions with a high density of functionally important elements and the strongest levels of purifying selection (Sankararaman et al. 2014; Vernot and Akey 2014; Sankararaman et al. 2016; Vernot et al. 2016). Moreover, the presence of “introgression deserts,” areas of the modern genome with exclusively AMH variants in modern populations, suggest that selection may have acted against gene flow in some regions (Vernot and Akey 2014; Sankararaman et al. 2016; Vernot et al. 2016). Some introgression deserts are found in regions with low recombination rates (Skov et al. 2020), consistent with neutral archaic alleles in the region being removed by purifying selection before they could recombine away from nearby deleterious alleles. Incompatibilities from epistasis that resulted in reduced male fertility, as evidenced by introgression deserts on the X chromosome and genes with higher expression in testes, may also explain some of the observed depletion in archaically introgressed alleles (Sankararaman et al. 2014; 2016; Telis et al. 2020), as could unannotated structural variation in archaic genomes (Vernot and Akey 2014). However, theoretical predictions suggest that most of the depletion of archaic introgressed haplotypes can be explained by purifying selection against weakly deleterious alleles (Harris and Nielsen 2016; Juric et al. 2016).

There has been some controversy over whether purifying selection against archaically introgressed deleterious alleles has acted gradually over long periods of time. Recently, Neanderthal ancestry has been shown to have not decreased significantly over the last 45,000 years (Petr et al. 2019), suggesting that purifying selection was strongest in the first few generations post introgression, consistent with theoretical expectations (Harris and Nielsen 2016; Juric et al. 2016). Selection has depleted Neanderthal alleles primarily in promoters, coding regions and conserved noncoding regions (Castellano et al. 2014). In contrast, Neanderthal alleles are enriched in gene expression-associated variants, suggesting that the functional impacts of Neanderthal introgression are more often through gene regulation than coding changes (Dannemann et al. 2017; Petr et al. 2019; Silvert et al. 2019). Some enhancer regions also show enrichment in Neanderthal alleles, such as adipose-related tissues and primary T cells (Dannemann et al. 2017; Petr et al. 2019; Silvert et al. 2019), but others, including brain and muscle-associated enhancers, show depletion (Telis et al. 2020). This is consistent with divergence between AMH and Neanderthal exomes in genes related to skeletal morphology, pigmentation, and behavioral traits (Castellano et al. 2014).

Forty-two types of tissues in humans show significant enrichment of Neanderthal variants in enhancers, with the highest rate of enrichment identified in adipose-related tissues and immune cells (Silvert et al. 2019). Additionally, there are several well-known examples of beneficial Denisovan and Neanderthal haplotypes that have been positively selected in modern human populations, including: the *EPAS1* locus related to hypoxia tolerance in Tibetans (Huerta-Sánchez et al. 2014; Racimo et al. 2017); the *BNC2* and *OCA2* loci related to skin pigmentation (Sankararaman et al. 2014; Vernot and Akey 2014; Gittelman et al. 2016); the *OAS* locus (Mendez et al. 2012; Gittelman et al. 2016; Sams et al. 2016), and Toll-like receptor loci (Gittelman et al. 2016; Dannemann and Kelso 2017) related to immune response; the *TBX15/WARS2* locus related to lipid metabolism in Inuit from Greenland and Native Americans (Huerta-Sánchez et al. 2014; Racimo et al. 2017); the *STAT2* locus related to innate immunity and found primarily in Melanesians (Mendez et al. 2012); and the *LARS* locus in Native Americans, which may be related to liver function (Racimo, Marnetto, et al. 2017). Finally, copy-number variations (CNVs), sections of the genome that are repeated a different number of times in different people, have also been adaptively introgressed from both Neanderthals and Denisovans near genes associated with metabolism, immunity, and development (Hsieh, et al. 2019). These results suggest that alleles relating to environmental pressures from high altitude (hypoxia tolerance), latitude and sun exposure (skin pigmentation), cold environments and dietary changes (lipid metabolism), and pathogens (immune response) increased in frequency after admixture, likely due to the important role they played in helping admixed human populations adapt to their environments. Moreover, most of these top candidate loci for adaptive introgression were not driven by the associative overdominance from recessive deleterious mutations, suggesting that they represent regions of true adaptation (Zhang, Kim, et al. 2020).

Adaptive introgression is particularly prominent around immune-related genes, suggesting that Neanderthals and Denisovans harbored many adaptive alleles to local pathogens that were positively selected after admixture with AMH. In particular, Enard and Petrov (2018) found that adaptively introgressed haplotypes are enriched for proteins that interact with RNA viruses. Similarly, polygenic adaptive introgression has been identified in pathways related to immunity (Gouy and Excoffier 2020). Moreover, population transcriptome studies of immune response to viral and bacterial pathogens in large numbers of cell cultures from individuals of European versus African ancestry have found many gene expression (Quach et al. 2016; Nédélec et al. 2016) and splicing (Rotival et al. 2019) differences that appear to be driven by Neanderthal introgressed alleles, providing further support for their regulatory impact on immunity.

**Fig. 3.**
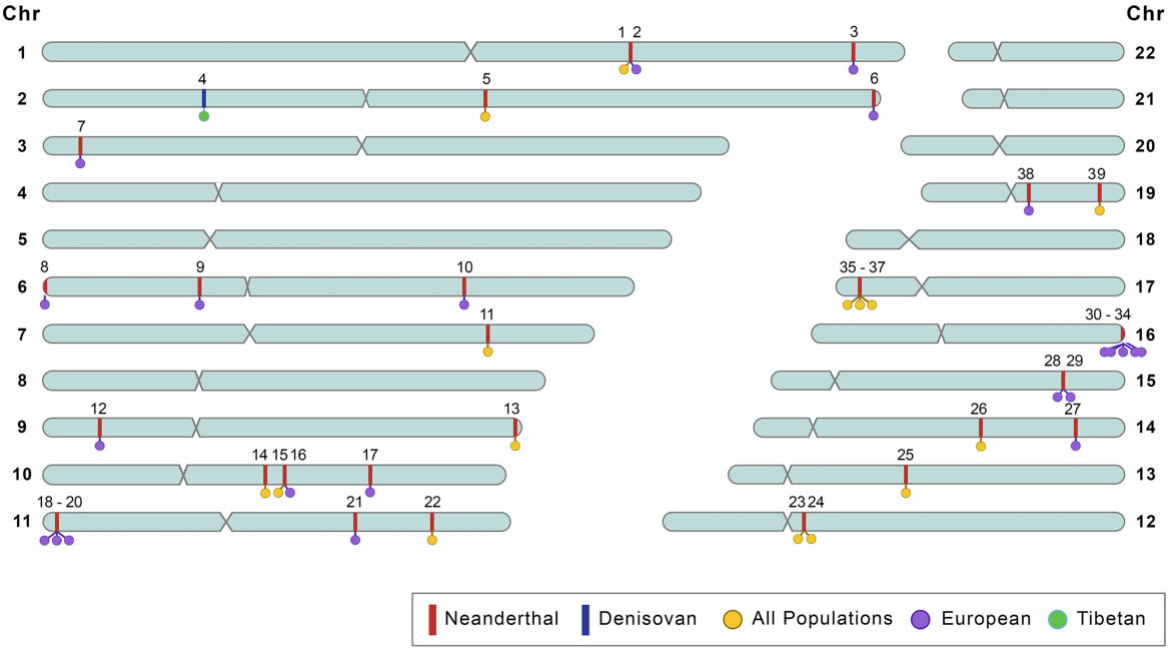
Distribution of a select subset of functionally associated SNPs of Neanderthal and Denisovan origin, and genes associated with functional phenotypes in the autosomes. Details on SNP-phenotype and gene–phenotype pairs shown in this figure can be found in table 2.

Recent studies have used a variety of methods to identify candidate alleles and regions underlying phenotypic impacts of archaic introgression (for a collection of examples, see table 2, fig. 3, supplementary table 1, Supplementary Material online, and github.com/SciFunk/data). Multiple studies have used large genotypic data sets with phenotypic data, such as electronic health records to link archaic alleles with specific traits, and have identified Neanderthal alleles associated with traits including neurological phenotypes, height, blood coagulation and inflammation, chronotype, and skin and hair pigmentation (Simonti et al. 2016; Dannemann and Kelso 2017; Prüfer et al. 2017). More recently, (McArthur et al. 2020) used an approach based on associating genome-wide trait heritability with Neanderthal ancestry to identify impacts of Neanderthal introgression on hair and skin, autoimmunity, chronotype, bone density, lung capacity, and menopause age. Although many associations between archaic alleles and phenotypes have been discovered, recent re-analysis has found that many of these associations are actually due to nonarchaic variants in linkage with archaic haplotypes (Skov et al. 2020). Many may actually be nonarchaic alleles shared between modern and archaic humans, still found in Africans today, that were lost in the OOA bottleneck but subsequently reintroduced in non-Africans by archaic introgression (Rinker et al. 2020). These last two studies highlight the complications in linking phenotypic impacts to archaic introgressed alleles.

**Table 2:** Select SNPs and Genes with Archaic Origin and Their Function Effects

	SNPs and Genes Linked to Phenotype	Reference		SNPs and Genes Linked to Phenotype	Reference
1	*SELP*, rs3917862, hypercoagulable state	Simonti 2016 ^N, E^	21	*CHORDC1*, skin color	Dannemann 2017 ^E^
2	*SLC35F3*, rs12049593, protein-calorie malnutrition	Simonti 2016 ^N, E^	22	rs1834481, interleukin-18 levels	Sankararaman 2014 ^N^
3	Increase in plasma prothrombin time (rs6013)	Skov 2020 ^N^	23	rs11175593, Crohn's disease	Sankararaman 2014 ^N^
4	*EPAS1*, hypoxia	Huerta-Sanchez 2014^D, T^	24	rs11564258, *MUC19*, Crohn's disease and inflammatory bowel disease	Rinker et al. 2020 ^N^
5	rs28387074, decreased concentration of hemoglobin	Skov 2020 ^N^	25	rs3118914, reduced height	Skov 2020 ^N^
6	*ASB1*, morning or evening person (chronotype)	Dannemann 2017 ^E^	26	rs72728264, decrease in mean corpuscular hemoglobin	Skov 2020 ^N^
7	*SLC6A11*, rs901033, tobacco use disorder	Simonti 2016 ^N, E^	27	*SLC24A4*, hair color (natural before graying)	Dannemann 2017 ^E^
8	*EXOC2*, hair color (natural before graying)	Dannemann 2017 ^E^	28	*ADAMTSL3*, impedance of leg (left and right)	Dannemann 2017 ^E^
9	*RUNX2*, skin color	Dannemann 2017 ^E^	29	*GOLGA6L4*, impedance of leg (left and right)	Dannemann 2017 ^E^
10	*GJA1*, pulse rate	Dannemann 2017 ^E^	30	*FANCA*, hair color (natural before graying)	Dannemann 2017 ^E^
11	rs12531711, systemic lupus erthematosus, primary biliary cirrhosis	Sankararaman 2014 ^N^	31	*SPIRE2*, hair color (natural before graying)	Dannemann 2017 ^E^
12	*BNC2*, ease of skin tanning, skin color, incidence of childhood sunburn	Dannemann 2017 ^E^	32	*TCF25*, hair color (natural before graying)	Dannemann 2017 ^E^
13	rs3025343, smoking behavior	Sankararaman 2014 ^N^	33	*MC1R*, hair color (natural before graying)	Dannemann 2017 ^E^
14	rs7076156 Crohn's disease	Sankararaman 2014 ^N^	34	*TUBB3*, hair color (natural before graying)	Dannemann 2017 ^E^
15	rs12571093, optic disc size	Sankararaman 2014 ^N^	35	rs75493593, type-2 diabetes	Sankararaman 2014 ^N^
16	*PBLD*, sitting height	Dannemann 2017 ^E^	36	rs75418188, type-2 diabetes	Sankararaman 2014 ^N^
17	*EXOC6*, daytime dozing or sleeping (narcolepsy)	Dannemann 2017 ^E^	37	rs117767867, type-2 diabetes	Sankararaman 2014 ^N^
18	*RHOG*, symptoms involving urinary system	Simonti 2016 ^N, E^	38	*ZNF536*, comparative height size at age 10 years	Dannemann 2017 ^E^
19	rs11030043, symptoms involving urinary system	Simonti 2016 ^N, E^	39	rs17632542, reduced risk of prostate cancer	Skov 2020 ^N^
20	*STIM1*, symptoms involving urinary system	Simonti 2016 ^N, E^			

Note.—All SNPs and genes have evidence for archaic introgression and functional effect. E—European, indicating that the SNP or gene was identified in a modern European population. N—Neanderthal, indicating that the source of the SNP or gene was a Neanderthal population. T—Tibetan, indicating that the SNP or gene was identified in a modern Tibetan population. D—Denisovan, indicating that the source of the SNP or gene was a Denisovan population. Citations without a modern population indicated (E or T) were detected using a broad panel of modern populations. Citations without Neanderthal or Denisovan indicated were detected using a method that generated a more general result of archaic introgression, without a specific population specified.

Similarly, the effects of archaic ancestry on gene expression in different tissues have also been studied. Using the Genome-Tissue Expression (GTEx) data set, McCoy et al. (2017) developed a method to estimate Neanderthal allele-specific expression in modern humans across tissues, and found a downregulation of Neanderthal allele expression in brain regions and testes. Colbran et al. (2019) used gene expression imputation models trained using GTEx to estimate expression differences between AMHs and Neanderthals, and found differences in genes associated with skeletal, cardiovascular, and immune functions. Finally, Dannemann et al. (2020) showed that existing induced pluripotent stem cell (iPSC) repositories contain iPSCs from European individuals with Neanderthal introgressed ancestry (Neanderthal Stem Cell Resource Browser), and demonstrated that single-cell transcriptomics of organoids generated from these iPSCs can be used to study the impact of Neanderthal introgressed alleles during development.

A major caveat to current phenotypic and gene expression analyses is that they primarily use data sets of European individuals, and therefore the majority of population-specific functional variants have been identified in Europeans (fig. 3). This limits the set of Neanderthal variants that can be studied, and currently precludes the study of many Denisovan variants. Moreover, models based on phenotype and transcriptome data from Europeans may not transfer to non-Europeans (Mikhaylova and Thornton 2019; Martin et al. 2019). Future studies of the impacts of archaic introgression will likely rely on expanded phenotypic, transcriptomic, and iPSC resources from more diverse populations; improved machine learning tools for unbiased regulatory effect prediction (Zhou et al. 2018; Jaganathan et al. 2019); and high-throughput functional and gene editing assays of variant effect (Findlay et al. 2018; Tewhey et al. 2018; van Arensbergen et al. 2019; Hanna et al. 2021). This will allow for a more complete analysis of the functional and phenotypic impacts of Neanderthal and Denisovan introgression across diverse populations.

## Conclusions

Over the past decade, exploration of the demographic history of AMH and archaic humans has demonstrated countless encounters between human populations. Encounters between AMH and archaic humans occurred at multiple geographic regions and at multiple time periods, even long before AMH ventured out of Africa. Archaic genomes themselves show evidence of gene flow between populations. In coming years, additional data sources and new methods will both play major roles in revealing more features of this complex and fascinating history. On the data side, sequencing additional high-coverage genomes of archaic individuals, particularly Denisovans, will be needed to better understand the genetic diversity present in archaic populations, and to uncover their demographic histories. We expect that analysis of large biobanks of tens to hundreds of thousands of individuals from multiple modern AMH populations (1000 Genomes Project Consortium et al. 2015; Sudlow et al. 2015; Skov et al. 2020) will provide access to more rare introgressed segments, which will allow us to better model archaic introgression events, and give us more insight into how introgression influences AMH phenotypes and health. There is an especially great opportunity to learn more about ancient and archaic populations originating in Africa, where relatively few modern individuals have been sampled (1000 Genomes Project Consortium et al. 2015; Malaspinas et al. 2016; Mallick et al. 2016; Skoglund et al. 2017; Henn et al. 2018; Bergström et al. 2020; Wang, Mathieson, et al. 2020; Sengupta et al. 2021), and where archaic remains are expected to remain scarce. Sequencing of additional individuals from all non-European populations remains a priority, in order to better understand the functional impact of archaic ancestry on a global scale.

In addition to sequencing archaic individuals and sampling from currently living populations, there is also great potential to increase sampling from ancient AMH individuals from throughout history and prehistory (Raghavan et al. 2014; Allentoft et al. 2015; Gallego Llorente et al. 2015; Malaspinas et al. 2016). As we increase the availability of these heterochronous samples we will both increase our confidence in some assertions about the past, and no doubt add additional layers of complexity and nuance to our understanding of the demographic history of these populations. With sufficient longitudinal data drawn from individuals living during different time periods around the world, we hope to see an increase in methods that place ancient and archaic individuals in a relative temporal and spatial context, and make better inferences about the structure of their populations, including gene flow from populations that cannot be sampled, such as ghost populations and superarchaic populations.

Although additional genomic samples will be one driver of discovery, a second important driver will be methodological and computational discoveries that enable more efficient data analysis and simulation. We predict that new statistical methods will be developed that take advantage of computationally efficient ARG summaries to infer features of archaic admixture. We also observe that efficient inference from simulation techniques are increasingly opening up new lines of inquiry, either using machine learning or methods like ABC, to perform inference without likelihood calculations, and with or without predefined summary statistics. Advances in existing ARG inference and phylogenetic methods will be required to take full advantage of new large scale and heterochronous samples.

Stemming from the combined power of additional data and methods to place data in context will come an unprecedented power to observe selective forces in action across human history. In coming years, we hope to see an increase in research that not only demonstrates the occurrence of admixture, but use new methods and heterochronous sampling to tie specific genetic variants to the process of adapting to new environments, or responding to the emergence of new selective pressures associated with disease, lifestyle changes, or natural disasters.

Fully exploring the implications of discoveries within the human genome will also require understanding changes outside the genome, in the environment and within the societies formed by AMH or archaic humans. Archaeological investigations into these questions are increasingly aided by ancient DNA found from environmental sources (Jørgensen et al. 2012; Willerslev et al. 2014; Zarrillo et al. 2018; Witt et al. 2021), or from the bones of wild and domesticated animals that lived alongside humans (Shapiro et al. 2004; Verdugo et al. 2019; Perri et al. 2021). Many methodological advances that will be first applied to humans and their close relatives we expect to quickly be extended to populations of animals and plants that also contain evidence of past events, and past environmental conditions faced by humans. Finally, there is hope on the horizon for new archeological techniques that can explore questions of genetic inheritance from protein sequencing alone, an advance that would allow for analysis of older samples and for analysis of samples where DNA has already been degraded (Welker 2018; Chen et al. 2019).

Although the last decade of innovation and research into the legacy of archaic admixture has been both incredibly promising and eventful, we fully expect the next decade to add even greater insight into the structure and complexity of our tangled family tree.

## Supplementary Material

Supplementary data are available at *Genome Biology and Evolution* online.

Box 1. Terminology Used in This Review to Address the Continuum of Hominin Species.

*Hominin:* Individuals assigned to the taxonomic subfamily Homininae. It includes all living humans and their ancestors, and all living chimpanzees, gorillas, and their ancestors, but excludes orangutans and their ancestors. Extinct members of *Australopithecus* and *Homo* are included in this subfamily.

*AMH:* Includes all living humans, and their ancestors, but excludes Neanderthals and Denisovans and their ancestors. The majority of AMH genomes coalesce to a single population living in Africa after the population split of AMH, Neanderthals, and Denisovans.

*Human*: Used here broadly to describe all individuals that contributed to the modern human gene pool.

*Archaic human:* Includes Neanderthals, Denisovans, and any other extinct human populations yet unsampled, but excludes AMH and their ancestors.

*Superarchaic human:* Any hypothetical human populations that diverged earlier than the population split of AMH, Neanderthals and Denisovans. Evidence of superarchaic ancestry has only been inferred indirectly and could correspond to any number of extinct populations of humans.

## Data Availability

No new data were generated or analyzed in support of this research.

## Supplementary Material

evab115_Supplementary_Data
