# Supplementary material for: Our Tangled Family Tree: New Genomic Methods Offer Insight into the Legacy of Archaic Admixture
Source: Genome Biol Evol. 2021 May 24;13(7):evab115. doi: 10.1093/gbe/evab115 (PMC8480178; doi:10.1093/gbe/evab115)
Supplement: evab115_Supplementary_Data [file evab115_supplementary_data.pdf]

Supplementary Table 1. All Genomic Locations and Effects of Archaic Introgression identified after 2013.

		Closest putative archaic source pop	Modern populations showing evidence of introgression or association	reference
	**SNP linked to phenotype**			
	rs12531711, systemic lupus erthematosus, primary biliary cirrhosis	Neanderthal		Sankararaman 2014
	rs3025343, smoking behavior	Neanderthal		Sankararaman 2014
	rs7076156, rs11175593, Crohn's disease	Neanderthal		Sankararaman 2014
	rs12571093, optic disc size	Neanderthal		Sankararaman 2014
	rs1834481, interleukin-18 levels	Neanderthal		Sankararaman 2014
	rs75493593, rs75418188, rs117767867, type-2 diabetes	Neanderthal		Sankararaman 2014
	SELP, rs3917862, hypercoagulable state	Neanderthal	European	Simonti 2016
	SLC35F3, rs12049593, protein-calorie malnutrition	Neanderthal	European	Simonti 2016
	RHOG, STIM1, rs11030043, symptoms involving urinary system	Neanderthal	European	Simonti 2016
	SLC6A11, rs901033, tobacco use disorder	Neanderthal	European	Simonti 2016
	loci associated with being treated with desloratadine, a drug used to treat allergies		European, East Asian	Steinrücken 2018
	rs11564258, MUC19, Crohn's disease and inflammatory	Neanderthal		Rinker

	bowel disease			2019
	reduced risk of prostate cancer (rs17632542), decreased concentration of hemoglobin (rs28387074), reduced height (rs3118914), decrease in mean corpuscular haemoglobin (rs72728264) and an increase in plasma prothrombin time (rs6013)	Neanderthal		Skov 2020
	**genes linked to phenotype**			
	EPAS1, hypoxia	Denisovan	Tibetan	Nielsen 2014
	SLC24A4,SPIRE2,TCF25,MC1R,TUBB3,FANCA: hair color (natural before graying)		European	Danneman 2017
	RUNX2, skin color		European	Danneman 2017
	BNC2, ease of skin tanning, skin color, incidence of childhood sunburn		European	Danneman 2017
	ZNF536, comparative height size at age 10 years		European	Danneman 2017
	GJA1, pulse rate		European	Danneman 2017
	ASB1, morning or evening person (chronotype)		European	Danneman 2017
	CHORDC1, skin color		European	Danneman 2017
	ADAMTSL3, GOLGA6L4, impedance of leg (left and right)		European	Danneman 2017
	PBLD, sitting height		European	Danneman 2017
	EXOC2, hair color (natural before graying)		European	Danneman 2017
	EXOC6, daytime dozing or sleeping (narcolepsy)		European	Danneman 2017
	**genes**			

	IL18, interleukin-18 levels	Neanderthal		McCoy 2017
	SVEP1, PLXNA2, ST5, RP1-283K11.2, UBE2H, TMEM9B, CTD-2003C8.2, PRSS45, RTN1, TRABD, TMEM9B, RP11-115J16.1, MMAB, AZIN1-AS1, TLR1, MAFB, UBE2H, CYB5A, PAPD7, RGL2, PABPN1L, LINC00693, PLEKHO1, MUC15, HSPG2, DDX60L, RP11-778H2.1, SNHG14, DNTTIP1, AC007389.3, KCNT2, TTBK1, SLCO1A2	Neanderthal		McCoy 2017
	TBX15, WARS2	Denisovan	Greenlandic Inuit, East Asians, Native Americans	Racimo 2017
	ANKRD27, MED15, PDGFRB, TGFBR2, IGF1, IGFBP2, IGFBP3, CXCR4, KLF3, PRRX1			Silvert 2019
	genes highly expressed in T-cells, including CXCR4,IL7R,IL10RA,NFKBIA,PTPRC			Silvert 2019
	regulatory alleles associated with HDHD5 (aka CECR5)	Neanderthal	Europeans	Rinker 2019
	**phenotypes**			
	phenotypes from electronic health records: actinic keratosis, mood disorders, depression, obesity, seborrheic keratosis, overweight, acute upper respiratory infections, coronary atherosclerosis	Neanderthal	European	Simonti 2016
	phenotype group: neurologic	Neanderthal	European	Simonti 2016
	facial morphology, body mass index, sleep, and metabolite levels in smokers	Neanderthal		Rinker 2019
	**GO term/category**			
	keratin filament	Neanderthal	European, East Asian	Sankararaman 2014
	phospholipid transporter activity, trace-amine receptor activity	Denisovan	Oceanian	Sankararaman 2016

	keratin filament	Neanderthal	American, Central/South/East Asian, West Eurasian	Sankararaman 2016
	cellular response to cadmium ion, cellular response to inorganic substance, cellular response to metal ion, cellular response to zinc ion	Neanderthal	Central, South, East Asian	Sankararaman 2016
	response to cadmium ion, cadmium ion binding	Neanderthal	South, East Asian	Sankararaman 2016
	chemokine-mediated signaling pathway, C-C chemokine receptor activity, chemokine receptor activity, cytokine receptor activity, G-protein coupled chemoattractant receptor activity	Neanderthal	South Asian	Sankararaman 2016
	cytokine production involved in inflammatory response, regulation of cytokine production involved in inflammatory response, extracellular region	Neanderthal	West Eurasian	Sankararaman 2016
	glycosphingolipid metabolic process, intracellular, intracellular membrane-bounded organelle, intracellular organelle, intracellular part, invadopodium, membrane-bounded organelle, organelle	Neanderthal	Oceanian	Sankararaman 2016
	intermediate filament, intermediate filament cytoskeleton	Neanderthal	American, Central Asian	Sankararaman 2016
	keratin, sensory perception (particularly olfaction)			Steinrücken 2018
	regulation of cell motility, insulin-like growth factor binding protein complex (for archaic alleles associated with genes expressed in adipose tissue)			Silvert 2019
